# Removal of Cd^2+^ from Water by Use of Super-Macroporous Cryogels and Comparison to Commercial Adsorbents

**DOI:** 10.3390/polym12102405

**Published:** 2020-10-19

**Authors:** Alzhan Baimenov, Dmitriy Berillo, Seitkhan Azat, Talgat Nurgozhin, Vassilis Inglezakis

**Affiliations:** 1 Environmental Science & Technology Group (ESTg), Chemical & Materials Engineering Department, School of Engineering & Digital Sciences, Nazarbayev University, Nur-Sultan 010000, Kazakhstan; alzhan.baimenov@nu.edu.kz; 2 Faculty of Chemistry and Chemical Technology, Al-Farabi Kazakh National University, Almaty 050012, Kazakhstan; seytkhan.azat@gmail.com; 3 Department of Pharmaceutical and Toxicological Chemistry, Pharmacognosy and Botany School of Pharmacy, Kazakh National Medical University, Almaty 050012, Kazakhstan; berillo.d@kaznmu.kz (D.B.); nurgozhin.t@kaznmu.kz (T.N.); 4 Faculty of Biology and Biotechnology, Al-Farabi Kazakh National University, Almaty 050012, Kazakhstan; 5 Institute of Chemical and Biological Technologies, Satbayev University, Almaty 050012, Kazakhstan; 6 Department of Chemical & Process Engineering, University of Strathclyde, Glasgow G1 1XJ, UK

**Keywords:** cryogels, adsorption, ion-exchange, complexation, cadmium, water treatment

## Abstract

In this study amphoteric cryogels were synthesized by the use of free-radical co-polymerization of acrylate-based precursors (methacrylic acid and 2-acrylamido-2-methyl-1-propansulfonic acid) with allylamine at different ratios. The physico-chemical characteristics of the cryogels were examined using SEM/EDX, FT-IR, XPS and zeta potential measurements. The cryogels were tested toward Cd^2+^ removal from aqueous solutions at various pH and initial concentrations. Equilibrium studies revealed a maximum sorption capacity in the range of 132–249 mg/g. Leaching experiments indicated the stability of Cd^2+^ in the cryogel structure. Based on kinetics, equilibrium and characterization results, possible removal mechanisms are proposed, indicating a combination of ion exchange and complexation of Cd^2+^ with the cryogels’ surface functional groups. The cryogels were compared to commercially available adsorbents (zeolite Y and cation exchange resin) for the removal of Cd^2+^ from various water matrices (ultrapure water, tap water and river water) and the results showed that, under the experimental conditions used, the cryogels can be more effective adsorbents.

## 1. Introduction

Heavy metals pollution can be harmful to humans and the environment. Cadmium (Cd) is a heavy metal commonly used in batteries, plastics and coatings production [1]. Cd stands out as a strong poison with a natural half-life of more than 20 years and in the human body it is strongly associated with metallothioneins [2]. As the main organ for the accumulation of toxic substances, the kidney is the main target tissue that exhibits early signs of intoxication [1]. Chronic manifestations of elevated Cd levels lead to liver damage, bone degeneration, blood damage, and renal dysfunction. The acceptable limit for cadmium in drinking water prescribed by the US Environmental Protection Agency is 0.005 mg/L [3].

The removal of heavy metals from water and wastewater is essential because of the numerous adverse effects on human health and the environment [1,4,5,6,7]. Several methods are available such as chemical precipitation [8], coagulation/flocculation [9], membrane filtration [10], ion-exchange [11], bioremediation [12] and adsorption [13]. Typical adsorbents such as activated carbons, resins, clays and natural and synthetic zeolites are commonly used, but they may exhibit low adsorption capacity, slow kinetics or require chemical treatment for the enhancement of their adsorption properties [14,15]. Frequently overlooked, removal kinetics are of paramount importance, especially when it comes to emergency situations when a rapid response is required. Therefore, candidate adsorbents must exhibit both high capacity and rapid removal kinetics.

An ideal adsorbent can exhibit several qualities such as; stable chemical structure, chelating sites to enhance selectivity or ion-exchange groups, hydrophilicity and three-dimensional (3D) network structure to maintain the water flow for a long time in filter applications, low cost production and facile synthesis method [16]. Polymeric materials such as cryogels and hydrogels can fit the abovementioned requirements. Macroporous hydrogels, also called cryogels, are hydrophilic sponge-like materials with developed macro- and super-porous polymeric matrices produced at sub-zero temperatures [17]. To increase the chelating capacity, useful for the selective elimination of heavy metal ions, various functional groups, such as carboxyl (-COOH), hydroxyl (-OH), amine (-NH_2_) or thiol (-SH), sulphogroup -SO_3_H, can be grafted onto cryogels’ surfaces. Cryogels exhibit several attractive properties such as fast adsorption kinetics, modifiability and high sorption capacity. Various monomeric compositions of hydrogels and cryogels have been used in the synthesis of macroporous polymers for Cd^2+^ removal. Table 1 summarizes the 3D polymeric adsorbents used for the removal of Cd^2+^ from water and their maximum removal capacity.

The aim if this work was to synthesize two cryogels and study them for the removal of Cd from ultrapure water. The cryogels were compared to commercial adsorbents (synthetic zeolite and ion exchange resin) and tested under different environmental conditions (tap water and river water). The pMAAc-DMAAm-AA-BisAAm and pAMPS-DMAAm-AA-BisAAm cryogel samples were prepared and named based on key monomers, methacrylic acid (MAAc) and 2-acrylamido-2-methyl-1-propansulfonic acid (AMPS) methylenebisacrylamide (BisAAm), respectively. Both compositions consist of acrylate-based precursors, allylamine and BisAAm cross-linking agent. The synthesized polymers were fully characterized and comprehensively studied for the removal of Cd^2+^ from aqueous solutions. To the best of our knowledge, this is the most detailed study on Cd^2+^ adsorption by using this kind of cryogels. Kinetics and equilibrium models were applied and in combination with post-sorption characterizations potential removal mechanisms are discussed. The Cd^2+^ removal studies from ultrapure, tap and river water and the comparison with commercial adsorbents provide valuable data on the performance of the adsorbents under real conditions.

## 2. Materials and Methods 

### 2.1. Materials

The basic monomers N,N-dimethylacrylamide (DMAAm, 99%), allylamine (AA) (98%), 2-acrylamido-2-methyl-1-propanesulfonic acid (AMPS), methacrylic acid (MAAc) (99%), cross-linking agent N,N-methylenebis (acrylamide) (BisAAm, 99%), 70% H_3_PO_4_, 5M NaOH, ammonium peroxodisulfate (APS, 98%) and N,N,Nʹ,Nʹ-tetramethyl ethylene diamine (TEMED, ≥99.5%) were used for synthesis of cryogels. The analytical purity Cd(NO_3_)_2_ (98%) from Sigma-Aldrich (Darmstadt, Germany) was used for all experiments. The water used for the preparation of all solutions was purified using a Puris MR-RO1600 (Mirae ST, Anyang, Korea) reverse osmosis unit. 

### 2.2. Synthesis of p(MAAc)-DMAAm-AA and p(AMPS)-DMAAm-AA Cryogels

The amphoteric cryogels, named p(MAAc)-DMAAm-AA and p(AMPS)-DMAAm-AA, were synthesized by free-radical polymerization technique using degassed ultrapure water as a solvent. The procedure of synthesis and quantities of the reagents used for p(MAAc)-DMAAm-AA and p(AMPS)-DMAAm-AA cryogels is discussed in detail elsewhere [28]. Briefly, pMAAc or pAMPS for p(MAAc)-DMAAm-AA and p(AMPS)-DMAAm-AA cryogels, respectively, and cross-linker BisAAm was added to preliminary degassed, by purging nitrogen gas, ultrapure water under vigorous stirring followed by acid neutralization by 5 M NaOH. Monomers DMAAm and AA were dissolved in degassed water under continuous stirring and acidified by adding concentrated H_3_PO_4_ to convert allylamine into salt. Subsequently, after mixing these two separately prepared solutions and dissolved oxygen removal, TEMED was added dropwise, mixed and cooled down to 2–4 °C for 30 min under nitrogen atmosphere followed by the addition of 5 wt% of APS under stirring. Finally, 2 mL of the monomeric mixture was poured into plastic syringes of 1 cm diameter. The syringes were rapidly sealed to avoid dissolution of oxygen from air in the solution and to prevent inhibition of radical polymerization. Then syringes were immersed in ethanol cooled cryobath having a program controlled refrigerated bath (Julabo F34, Seelbach, Germany) and kept at −12 °C for 24 h. The obtained monolithic cryogels were thawed out in warm water (23–25 °C) and washed firstly with 10% ethanol and then with 2 L of pure water. Further characterization and experiments of both types of cryogels were freeze-dried on FreeZone 2.5 L (Labconco, Kansas City, MO, USA) instrument via lyophilization technique at −45 °C and under vacuum (0.4 mbar) for 48 h in order to remove water.

### 2.3. Materials Characterization

The morphological characteristics of the cryogels were studied by using a Zeiss Crossbeam 540 scanning electron microscope (SEM) at 3–10 kV, equipped with a backscattered electron detector. Spot (point) and area (mapping) elemental analysis was carried out using an energy-dispersive X-ray (EDX) spectrometer (INCA X-sight, Oxford Instruments, High Wycombe, UK) connected to the SEM.

For determination of functional groups, the lyophilized and crushed into fine powder samples were analyzed using FT-IR. Infrared spectra were recorded for the sum of 32 scans at a resolution of 4 cm^–1^ in the range of 4000–400 cm^–1^ using the Agilent Technologies Cary 600 Series FTIR spectrophotometer equipped with diamond ATR.

The surface charge of crushed samples was evaluated via zeta potential measurement by batch equilibration method. A mass of 10 mg of fine powder of the polymer was equilibrated with 10 mL of aqueous solution at initial pH from 2 to 9 values adjusted by adding an appropriate amount 0.1 M HCl and 0.1 M NaOH keeping the ionic strength constant, for 24 h by shaking at room temperature. A Zetasizer Nano (Malvern, Malvern, UK) was used to determine the zeta potential of cryogels from the electrokinetic data (zeta potential vs. pH).

The X-ray photoelectron spectroscopy (XPS) measurements were conducted on a VG-Microtech Mutilab 3000 device equipped with a 9 channeltrons hemispherical electron analyzer and X-ray radiation source with Mg and Al anodes. The binding energies (BE) were calibrated by a C1s core level at 284.8 eV as a reference.

### 2.4. Effect of pH

To evaluate the effect of pH on the adsorption efficiency of the cryogels, a set of experiments were performed in a range of pH 1.0–5.0 at room temperature using 0.07 g of the polymers in 100 mL of 200 ppm metal ion solutions. The pH of Cd^2+^ solutions with initial concentration of 200 ppm was adjusted by HNO_3_ to reach desired pH values. The residual concentrations of metal ions were analyzed by iCAP RQ ICP-MS analyzer (Thermo Scientific, Waltham, MA, USA).

### 2.5. Batch Adsorption Kinetics

The Cd^2+^ solutions were prepared by dissolving analytical grade Cd(NO_3_)_2_ in ultrapure water. A volume of 100 mL of 100 mg/L Cd^2+^ solution without adjustment (pH 5.82) or adjusted to pH 4 was mixed with 0.07 g of cryogel in plastic tubes under shaking at 120 rpm at room temperature. At pre-determined time intervals, 0.1 mL of sample was taken from the tubes and analyzed by ICP-MS (iCAP RQ, Thermo Scientific, Waltham, MA, USA) with appropriate dilution. The total sampling volume was kept lower than 3% of the initial volume in all experiments. Blanks with the same initial concentration of metal ions and volume without solids were measured. Blank experiments showed that a Cd^2+^ loss due to adsorption on tube walls was less than 2%. All experiments were carried out in duplicate and the experimental standard deviation was less than 5%. The amount of adsorbed Cd^2+^ was calculated by difference of concentrations between initial and final solutions, expressed on a cryogel mass as it shown in Equation (1):

(1) $$q_{\mathrm{eq}}=\frac{C_{o}-C_{f}}{m}\times V$$

where q_eq_ is the amount of Cd^2+^ ions adsorbed (mg/g), C_o_ and C_f_ are Cd^2+^ ion concentrations (mg/L) in the initial and final solutions, respectively, V the volume of solution (L) and m is the weight of the cryogel (g). To avoid misunderstanding, throughout the paper the term “loading” and symbol q (mg/g) is used for the amount of cadmium adsorbed per initial weight of the cryogel (before adsorption), while the term “content” and symbol ct (mg/g) for the amount of cadmium adsorbed per total weight of the solid phase (initial weight of cryogel plus the weight of the adsorbed Cd^2+^). The loading is typically used for kinetics and equilibrium studies, whereas the content for EDX analyses and are related as follows (Equation (2)) [29]:

(2) $$\mathrm{ct}=\frac{q}{1+\left(\frac{q}{1000}\right)}$$

The released sodium ions from macroporous hydrogels during cadmium removal were determined by Dionex ICS 6000 ion-chromatography instrument (Thermo Scientific, Waltham, MA, USA). The release of sodium ions from cryogels in the absence of metal ions were done by placing 80 mg of dry cryogel in 100 mL of ultra-pure water under shaking at 120 rpm for 30 days. The total release of Na^+^ ions from both types of cryogels was no more than 1.3 mg/g.

### 2.6. Equilibrium Adsorption Isotherms

Equilibrium adsorption isotherms were obtained in the batch mode without pH adjustment. Three different isotherm adsorption experiments were conducted: experiment 1 (exp#1)—a mass of cryogel between 0.0022 and 0.150 g was mixed with 100 mL of Cd^2+^ solution of constant initial concentration at 100 mg/L; experiment 2 (exp#2)—a constant mass of cryogel (0.07 g) was mixed with 100 mL of Cd^2+^ solution with a concentration ranging from 100 to 1000 mg/L and experiment 3 (exp#3)—a mass of cryogel between 0.07 to 0.9 g was mixed with 100 mL of Cd^2+^ solution of constant initial concentration at 1000 mg/L. All experiments were done in plastic tubes under shaking by use of a Rotamax 120 (Heidolph, Schwabach, Germany) shaker at room temperature until equilibrium was reached. The attainment of equilibrium was monitored by sampling and measuring the residual Cd^2+^ daily until no concentration change was observed. The total liquid sampling volume was less than 3%. The experiments were conducted at least in duplicate. The average relative standard deviation for all experiments was 1.8% while on graphs the absolute standard deviation values are reported for each solid phase loading.

Taking into account the unavoidable analytical error, it is recommended that sorption experiments are designed in such a way so that the difference between the aqueous phase initial and equilibrium concentrations is not too small, otherwise the error in the calculated solid phase equilibrium loading may become very high and the results unreliable (see Equation (1)). This may happen when a small amount of adsorbent is used, leading to high aqueous phase equilibrium concentration and thus a small difference with the aqueous phase initial concentration. For instance, for a relative analytical error in concentration of around 2.5% and a desired relative error in capacity below 15% the aqueous phase equilibrium concentrations must be kept below 85% of the initial concentration. In an effort to avoid extensive uncertainties this rule was followed in the present study.

### 2.7. Leaching Experiments 

The evaluation of the retention of the Cd^2+^ on the amphoteric cryogels after adsorption was studied by leaching experiments under pH 6.5. First, the cryogel samples that were used in the equilibrium adsorption isotherms experiments with the highest loading were washed with 1 L of ultrapure water to wash out the residual solution in the structure tightly closed containers were left for 30 days under shaking at 120 rpm at room temperature. The samples were withdrawn from each container and analyzed for leached Cd^2+^. All the leaching experiments were carried out in duplicate and the average values are reported.

### 2.8. Removal from Various Water Matrices by Amphoteric Cryogels and Commercial Adsorbents

The adsorption studies were conducted in batch mode by mixing a certain volume of 200 mg/L Cd^2+^ solution with different water matrices to a final volume of 50 mL and concentration of 20 mg/L. Ultrapure water (Puris MR-RO1600, Mirae ST, Anyang, Korea), tap water (from Nazarbayev University labs) and river water (sampled from 43°15’4.0”N 76°51’50.7”E, Bolshaya Almatinka, Almaty, Kazakhstan) were used without any further purification. The cryogels were comparedwith commercial ion exchange materials, namely an H⁺-form ion exchange resin (Merck, Darmstadt, Germany) and sodium Y synthetic zeolite (Sigma Aldrich, Darmstadt, Germany). A mass of 0.1 g of each adsorbent was mixed with 50 mL of 20 mg/L Cd^2+^ solutions at room temperature. Samples were withdrawn at 30 min, 2 h and 24 h and the residual Cd^2+^ was measured by a Dionex ICS 6000 ion chromatography system (Thermo Scientific, Waltham, MA, USA) after filtering through 0.45 µm hydrophilic filter and dilution. The experiments were done in duplicate and average values are reported. The average standard deviation was less than 3%.

## 3. Results and Discussion

### 3.1. Synthesis and Characterization of Amphoteric Cryogels

**Figure 1:**
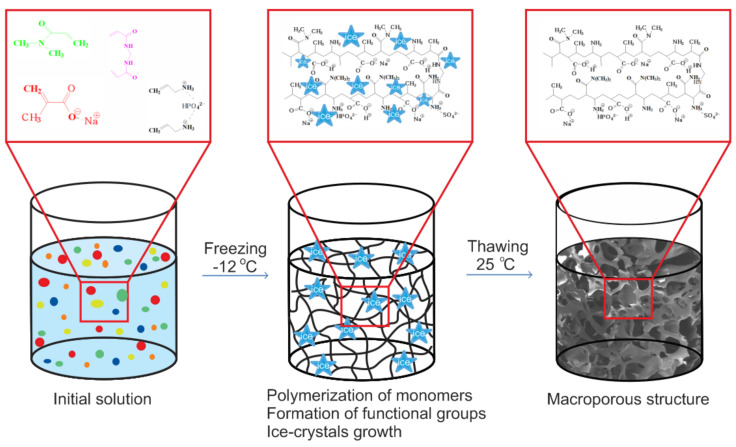
Schematic representation of the formation of p(MAAc)-DMAAm-AA cryogel.

To visualize the mechanism of polymerization at subzero conditions the cryopolymerization reaction of formation of polymeric chain occurring around the developing ice-crystals the example of p(MAAc)-DMAAm-AA cryogel preparation is presented on the Figure 1. The methacrylic acid co-polymerized with allylamine gives a low yield of the gel fraction due to the low activity of allylamine [29]. One way to enhance the activity of allylamine is to convert it to a phosphate complex [30,31], which can be done via mixing of allylamine with phosphoric acid. Simultaneously to radical polymerization of selected monomers and the crosslinking of polymer chains takes place to form branched macromers, ice crystals grow, which displace all components of the reaction mixture into an unfrozen liquid microphase [29]. The process of polymerization goes on until free monomers run out or until termination of all radicals. Upon completion of the polymerization reaction and ice thawing, a hollow of microcrystals leads to macro-sized pores development within the polymeric structure.

The formation of various functional groups during the polymerization step in the structure of cryogels was recorded by FT-IR analysis whose spectra are shown in Figure S1. The FT-IR shows that both materials have peaks at 3320–3286 cm^−1^, which are ascribed to the stretching vibration bands of OH- , which overlaps with N-H group in the same region, while ~2920 cm^−1^ corresponds to sp^3^ hybridization of –CH –CH_2_, –CH_3_ groups. The absorption bands at around ~1620 and ~1540 cm^−1^ are attributed to C=O stretching of amide(I) and NH-bending of amide(II) groups, respectively [32]. Both cryogels show the existence of amide(I) and amide(II) groups due to the presence of pDMAAm and BisAAm reagents in the structure of cryogels. Furthermore, the peak around 1390 cm^−1^ is assigned to the COO^-^ carboxyl groups of amino acid residues [33]. Non-dissociated carboxylic groups at 1392 cm^−1^ and stretching vibrations bands of a dissociated carboxyl group at 1141 cm^−1^ are observed in p(MAAc)-DMAAm-AA cryogel [34]. On the other hand, the adsorption peaks at around 1143–1036 cm^−1^ probably correspond to phosphate groups [33] due to the use of H_3_PO_4_ in synthesis for both cryogels. Inasmuch as p(AMPS) polymer contains 2-acrylamido-2-methyl-1-propanesulfonic acid monomer in the structure, the characteristic frequencies of sulfonic acid, sulfoxide, sulphide and C-S functional groups appear at 1452–1401 cm^−1^, 1178 cm^−1^, 1036 cm^−1^ and 621 cm^−1^, respectively [35,36,37].

The zeta potential measurements results are presented in Figure S2. The surface charge of p(MAAc) cryogel in a wide range of pH from 3.4 to 9 is negative because of deprotonation of carboxyl groups. A positive charge of p(MAAc)-DMAAm-AA at pH below 3.2 is attributed to positively charged aminogroups of allylamine and therefore at pH below 3.2 mostly negatively charged ions will be adsorbed but adsorption of cations via ion exchange mechanism is also possible [38]. In case of p(AMPS)-DMAAm-AA cryogel surface is negatively charged for the whole pH range due to the presence of permanently negatively form of dissociated sulfonic acid, which is a strong acid. It can be also assumed that aminogroups of allylamine and neighboring sulfonic and carboxyl groups are protonated at low pH.

**Figure 2:**
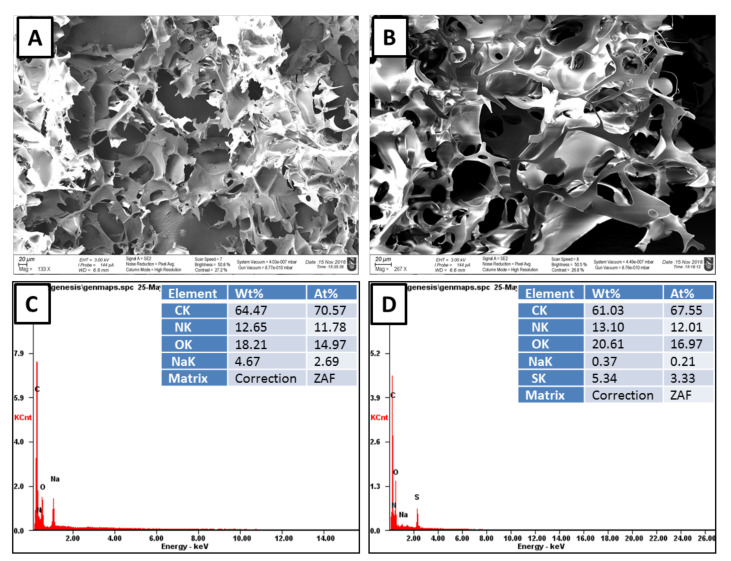
SEM and elemental composition according to point EDX analysis of (**A**,**C**) the p(MAAc)-DMAAm-AA and (**B**,**D**) p(AMPS)-DMAAm-AA cryogels.

The skeletal morphology of the cryogels was examined by scanning electron microscope. SEM microimages showed an interconnected network incorporated with super-macropores with pore diameters from 10 to 100 μm (Figure 2A,B).

SEM/EDX analysis is often used for elemental composition of cryogels [28,38]. The mass percentage of carbon, oxygen and nitrogen were found to be in approximately the same level for p(MAAc)-DMAAm-AA and p(AMPS)-DMAAm-AA cryogels. NaOH used for neutralization of p(MAAc)-DMAAm-AA cryogel led to the mass percentage of Na in the final product of 4.67%. p(AMPS)-DMAAm-AA cryogel the main monomer was sulfur-containing monomer AMPS and the amount of sulfur in the adsorbent was equal to 5.34% (Figure 2C,D).

### 3.2. Adsorption Kinetics 

**Figure 3:**
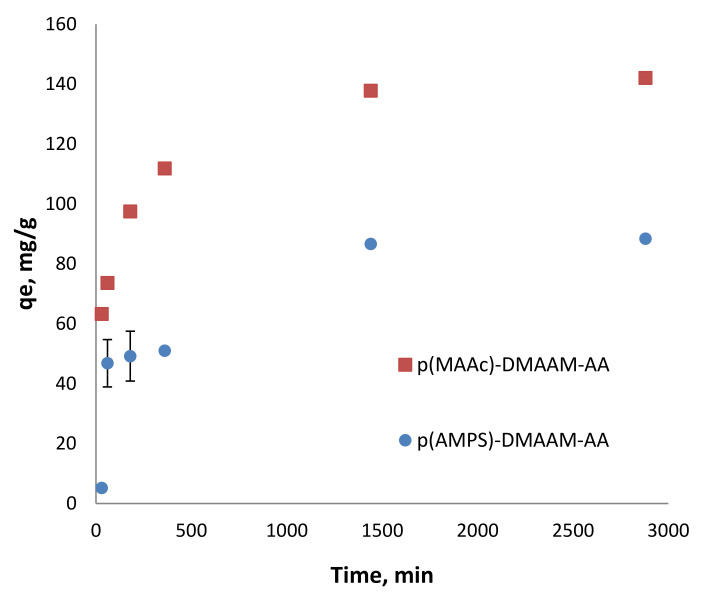
Cd^2+^ removal kinetics on the p(MAAc)-DMAAm-AA and p(AMPS)-DMAAM-AA cryogels (70 mg of cryogel in 100 mL of solution) at pH 5.5.

The effective and fast removal of highly toxic metal ions is of great interest, especially in emergency when a rapid response is required. To evaluate the sorption kinetic of Cd^2+^ ions on cryogels, batch experiments at pH 4 and 5.82 were conducted. The difference in pH did not play a significant role in the kinetics of cryogels for Cd^2+^ since the initial pH of solutions was higher than the isoelectric point of cryogels [29]. As the kinetics was similar at both pH values, in the range of the experimental error, only the results at pH 5.82 are shown in Figure 3. In the first hour p(MAAc)-DMAAm-AA cryogel was able to remove more than 50% from the metal ions solution while p(AMPS)-DMAAm-AA removed only about 32.5%. The p(MAAc)-DMAAm-AA sample removed almost 98%, while p(AMPS)-DMAAm-AA cryogel reached the maximum removal at 60% after 24 h of adsorption.

Two commonly used simplified kinetics models were used to further study the mechanisms of removal. The pseudo-first-order (Equation (3)) and pseudo-second-order models (Equation (4)) [39,40] were used in linear forms expressed as follows:

(3) $$\ln\left(q_{e}-\;q_{t\;}\right)=\ln q_{e}-k_{1}t.\;$$

(4) $$\frac{t}{q_{t}}=\frac{1}{k_{2\;}q_{e}^{2}}+\;\frac{t}{q_{e}}$$

where *q_e_* and *q_t_* are amounts (mg/g) of Cd^2+^ ion adsorbed at equilibrium and at specific time t (min), respectively; *k_1_* (min^−1^) and *k_2_* (g mg^−1^ min^−1^) are the pseudo-first-order and pseudo-second-order constants. The constant k_1_ and the q_e_^cal^ (calculated equilibrium loading) can be obtained from the slope and intercept of the linear plot of ln(q_e_^exp^ − *q_t_*) versus *t*, where q_e_^exp^ is the experimental equilibrium loading. The values of *k_2_* and q_e_^cal^ can be calculated from the linear fit of *t/q_t_* versus *t*. The results of kinetic models are given in Table 2.

The obtained results for both p(MAAc)-DMAAm-AA and p(AMPS)-DMAAm-AA cryogel reveal that the linear correlation coefficients (R^2^) of the pseudo-second-order model are close to 1 (0.98–0.99). Additionally, the calculated equilibrium loading values (q_e_^cal^) of the pseudo-second-order kinetics better fits the experimental equilibrium loading (q_e_^exp^) with a maximum error of 3 and 11% for p(MAAc)-DMAAm-AA and p(AMPS) cryogel, respectively.

**Figure 4:**
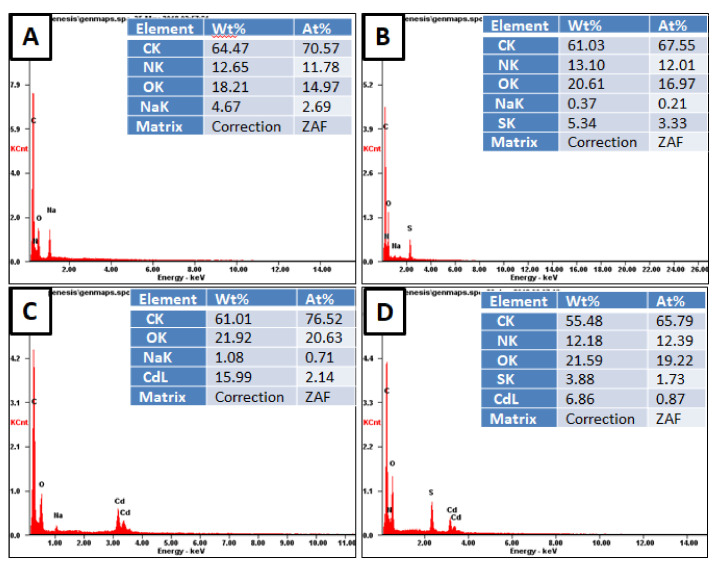
Elemental composition of the cryogels before and after interaction with metal ions according to spot EDX analysis (**A**) p(MAAc)-DMAAm-AA, (**B**) p(AMPS)-DMAAm-AA, (**C**) p(MAAc)-DMAAm-AA-Cd and (**D**) p(AMPS)-DMAAm-AA-Cd.

The existence of adsorbed Cd^2+^ metal ions on the cryogels surface was proved by point EDX analysis and is presented in Figure 4. Eventhough the EDX technique is semi-quantitative it gives a content (ct) of 159.9 and 68.6 mg/g for p(MAAc)-DMAAm-AA and p(AMPS)-DMAAm-AA, respectively, which is reasonably close to the content calculated from the equilibrium data (Table 2 and Figure 4). Furthermore, the elemental analysis confirms that p(MAAc)-DMAAm-AA cryogel has higher adsorption capability compared with p(AMPS)-DMAAm-AA sample. The conducted EDX mapping analysis images reveal that adsorbed metal ions are evenly distributed over the entire surface of the polymers (Figures S3 and S4). The post-adsorption EDX analysis of cryogels showed that no nitrogen exists on the p(MAAc)-DMAAm-AA cryogel, while before adsorption the nitrogen content was 12.65% (Figure 4a). This can be explained by the involvement of amino groups in the complexation reaction, as a result of which metal ions makes the detection of smaller nitrogen ions more difficult in EDX analysis. [38]. Furthermore, after the interaction with Cd^2+^, the EDX technique did not detect any Na, which is an indication of the ion-exchange mechanism.

### 3.3. Effect of pH on Metal Ions Adsorption

**Figure 5:**
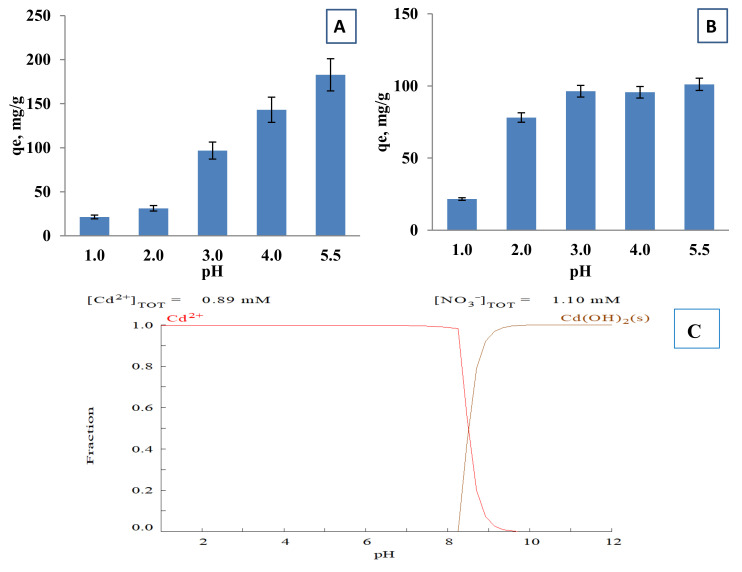
Equilibrium loading of Cd^2+^ ions by (**A**) p(MAAc)-DMAAm-AA and (**B**) p(AMPS)-DMAAm-AA cryogels and (**C**) the speciation of cadmium ions at different pH values.

The pH is one of the most influential properties affecting the adsorption and ion exchange of metal ions. In alkaline environment metal ions form hydroxide complexes, hindering their removal from the aqueous phase and form precipitates rendering the sorption studies difficult. At the acidic medium at pH 1, the Cd^2+^ ions solid phase equilibrium loading on both cryogels was low at about 21 mg/g (Figure 5). With the rise of the pH to 3 the equilibrium loading capacity increased almost 5–7 times in comparison with values at pH 1. The removal of the Cd^2+^ is gradually increasing with an increase of the initial pH from 3 to 5 with an equilibrium loading of 182.8 mg/g by p(MAAc)-DMAAm-AA and 101.1 mg/g of p(AMPS)-DMAAm-AA. The speciation of Cd^2+^ at different pH values was simulated by Medusa software and is presented at Figure 5C. As is evident, cadmium is in the form of Cd^2+^ up to pH 8, exhibiting simple speciation, and as hydrogen ions act as competitors for the ion exchange sites Cd^2+^ removal is higher at higher pH. Another factor is the surface charge (Figure S2), which in low pH is positive resulting in ionic repulsion of the metal ions. Finally, at higher pH values, redundant weak and strong acidic carboxylic and sulfonate groups, respectively, may provoke strong electrostatic interactions of the metal ions with the negatively charged functional groups of cryogels, which improves the adsorption capacity [41].

### 3.4. Effect of Metal Ions Initial Concentration and Cryogels Loading for Adsorption Isotherms 

**Figure 6:**
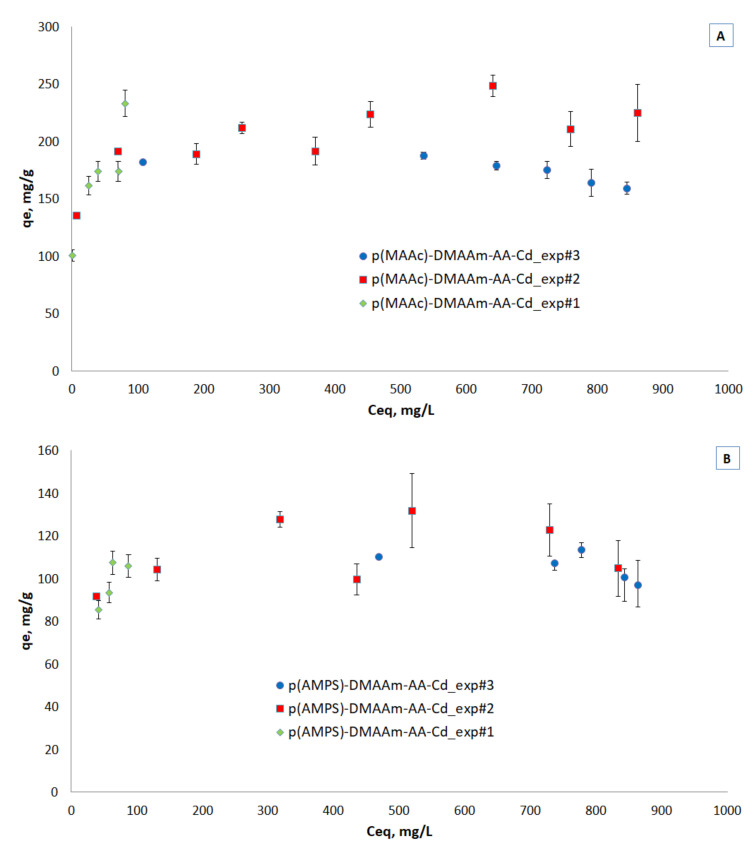
Isotherms of metal ions removal by (**A**) p(MAAc)-DMAAm-AA and (**B**) p(AMPS)-DMAAm-AA cryogels.

Three equilibrium adsorption isotherms for both cryogels are presented in Figure 6A,B. The equilibrium studies were done by different methods, namely by varying the mass of solids under constant solution volume and concentration at 100 mg/L (exp#1) and 1000 mg/L (exp#3), which is a common method in ion exchange studies and by varying the initial concentrations under constant solids mass and solution volume (exp#2), which is a common method in adsorption studies. This kind of study is rare and can provide insights into the removal mechanisms. 

Despite the variation in high liquid phase equilibrium concentrations, the three methods seem to result in a single isotherm for both materials, reaching a plateau at low liquid phase equilibrium concentrations, similar to Type I adsorption isotherm [42]. The discrepancies at high liquid phase equilibrium concentrations can be attributed to the contribution of ion exchange. As is well known, the ion exchange isotherms for the case of exchange of ions of different valence, depend on the initial concentration [41]. This effect is interesting to be studied in more detail but is out of the scope of the present study. The capacity of the p(MAAc)-DMAAm-AA cryogel reached 249 mg/g and 132 mg/g of p(AMPS)-DMAAm-AA cryogel. 

Being the most frequently used experimental method for the derivation of adsorption isotherms the results of the exp#2 were used for fitting the experimental data with the Langmuir and Freundlich models. The linear form of the Langmuir sorption isotherm [43] is (5):

(5) $$\frac{C_{e}}{q_{e}}=\frac{1}{q_{m}K_{L}}+\;\frac{C_{e}}{q_{m}}R_{L}=\frac{1}{1+K_{L}C_{0}}.\;$$

where *C_e_* (mg/L) is the equilibrium concentration of Hg^2+^ ions in solution, *q_e_* and *q_m_* are the equilibrium and maximum adsorption capacities in mg/g, whereas *K_L_* is the Langmuir constant (L/mg). The RL value is a dimensionless constant of Langmuir model ratifies the adsorption process to be linear (RL = 1), favorable (0 < RL < 1), unfavorable (RL > 1) or irreversible (RL = 0). The linear form of the Freundlich sorption (6) isotherm is [29]:

(6) $$logq_{e}=logK_{F\;}+\frac{1}{n}logC_{e}$$

where *C_e_* (mg/L) and *q_e_* (mg/g) is the concentration and adsorption capacity at the equilibrium and *n* (dimensionless) and *K_F_* constants (units depend on the *1/n*). The linearized isotherm plots according to Langmuir and Freundlich are presented in Figure S5a-d for both cryogels.

From the results of used models (Table 3) it is evident that the Langmuir isotherm model fits the experimental results better for both cryogels and the calculated capacities are close to the experimental. The adsorption capacities of both p(MAAc)-DMAAm-AA and p(AMPS)-DMAAM-AA cryogels toward Cd^2+^ are high and comparable to other 3D polymeric materials reported in the literature. Godiya et al. fabricated carboxymethyl cellulose/polyacrylamide composite hydrogel for removal of Cd^2+^, Pb^2+^ and Cu^2+^ ions from aqueous solutions. The maximum removal capacity of Cd^2+^ was 256 mg/g at pH 5.5 and initial metal concentration 100 ppm [16]. Elgueta et al. developed hydrogels using hydroxyethylmethacrylate (HEMA)-AMPS co-polymerized with various initiators and cross-linking agents for adsorption of different heavy metal ions [19]. In this study samples reached maximum adsorption capacity values of 226 mg/g at pH 5.0. The authors state that at pH 5.0 the sulfonate groups of polymers protonated and have high binding affinity toward cations through electrostatic interaction followed by ion-exchange process.

### 3.5. Mechanisms of Removal

**Figure 7:**
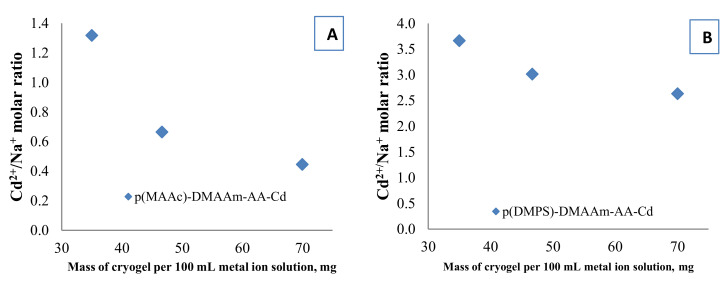
Cd^2+^/Na^+^ molar ratio in selected experiments for (**A**) p(MAAc)-DMAAm-AA-Cd and (**B**) p(AMPS)-DMAAm-AA-Cd (right) samples

The functional groups of amphoteric macroporous hydrogels can act as ion exchangers and also allow chemical reactions rendering the removal mechanism of Cd^2+^ complex. One of the proposed removal mechanisms is the ion exchange process between H^+^, Na^+^ or both, of carboxylic and sulfo groups in the cryogel structure with metal ions from the solution phase followed by complexation of Cd^2+^ with functional groups. To support this hypothesis Na^+^ ions released after the interaction of Cd^2+^ with cryogels were measured. In the case of a purely ion exchange mechanism between Cd^2+^ and Na^+^, the Cd^2+^/Na^+^ molar ratio must be 0.5. The Cd^2+^/Na^+^ molar ratio is shown in Figure 7 and depends on the mass of the cryogel/solution volume ratio. In the case of p(MAAc)-DMAAm-AA cryogel the molar ratio is around 0.5 for some experiments, which is evidence of ion exchange between Cd^2+^/Na^+^. In the case of p(AMPS)-DMAAm-AA cryogel, the Cd^2+^/Na^+^ ratio is always higher than 0.5 which means that the released amount of Na^+^ is small. This happens due to the low initial concentration of Na^+^ in the p(AMPS)-DMAAm-AA cryogel and probably also because of the sulfonic acid’s H^+^, which might be exchanged for Cd^2+^.

**Figure 8:**
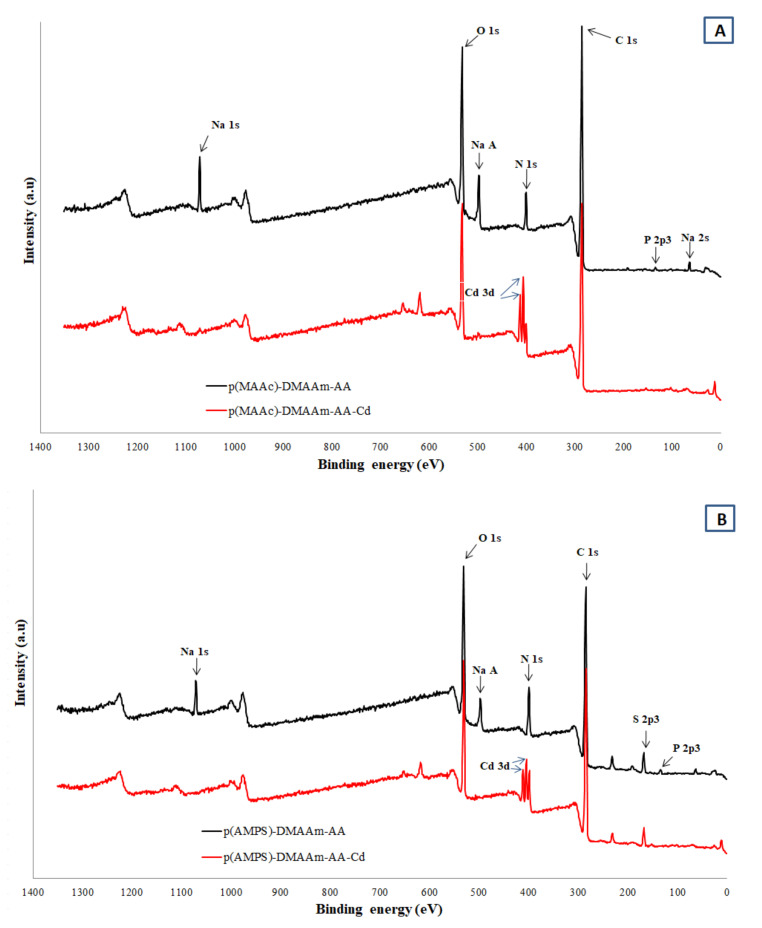
XPS patterns of cryogels before and after metal ions removal (**A**) p(MAAc)-DMAAm-AA (**B**) p(AMPS)-DMAAm-AA cryogels.

For more in depth investigation of adsorption mechanism and study the cryogel-Cd^2+^ interaction the XPS characterization was conducted before and after adsorption (Figure 8a,b). In the p(MAAc)-DMAAm-AA cryogel before adsorption, the Na 1S peak at ~1071.4, Na Auger peak ~497.1 and Na 2s peak at ~65.1 eV were clearly identified. After the interaction with Cd^2+^ the Na peaks disappeared with the advent of corresponding to Cd peaks at 3d_5/2_ and 3d_3/2_ at 405.6 and 412.3 eV indicating the contribution of ion exchange in the removal of Cd. The C 1S peaks of p(MAAc)-DMAAm-AA cryogel at 284.6, 286.8 and 288.2 eV correspond to C-C, C-O and C=O chemical states of carbon [44]. After the interaction of both cryogels with Cd^2+^, the binding energy of C-O and C=O are shifted for 0.7–1.2 eV to higher positions due to the binding of positively charged Cd^2+^ via donor acceptor interactions of electrons of oxygen with vacant orbital of cadmium. The N 1s core levels are centered at 399.9 and 401.6 eV, which can be assigned to the non-protonated and protonated amine/amide groups [33]. Moreover, in the structure of parent p(MAAc)-DMAAm-AA the small peak of HPO_4_^2–^/PO_4_^3–^ is found at the 133.2 eV while after the interaction with Cd^2+^ this peak disappeared, because of substitution phosphate anion counter ion of protonated aminogroup on other counter ion or deprotonation of aminogroup -NH_2_. Figure 8b shows the spectrum of p(AMPS)-DMAAm-AA cryogel before and after metal ions removal. The S 2p core level spectrum is deconvoluted into two major peaks centered at 167.5 and 169 eV, which are assigned to 2 p_3/2_ and 2 p_1/2_ oxidized states of sulfur corresponding to sulfonic acid group (-SO^3–^) [45]. After the interaction of Cd^2+^ the sulfonic acid group peaks are shifted by 0.5–0.7 eV to higher energies which suggests the formation of coordination bonds of sulfonic acid residues with Cd^2+^. These findings indicate the involvement of the carboxylic and sulfonic groups in the chelation process, which resulted in the high adsorption capacity of the cryogels.

**Figure 9:**
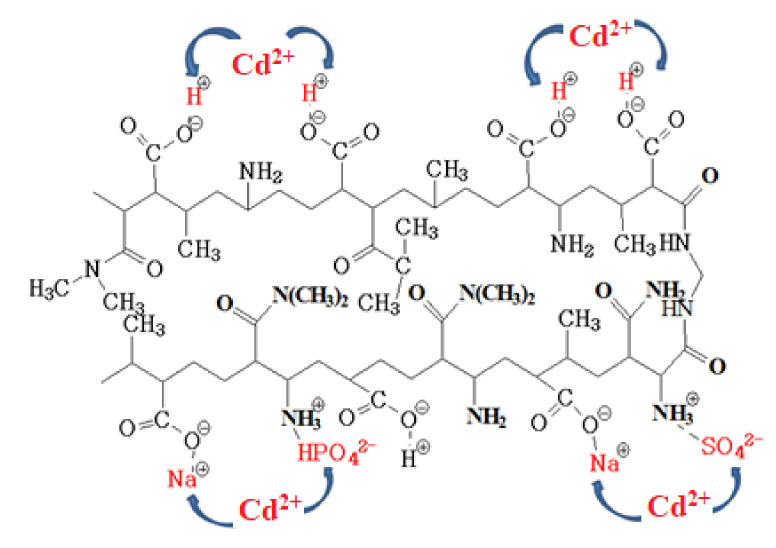
Proposed complexation of Cd^2+^ with functional groups of p(MAAc)-DMAAm-AA.

The proposed mechanism of Cd^2+^ removal is presented in Figure 9 for p(MAAc)-DMAAm-AA as an example.

### 3.6. Leaching Experiments 

The cryogels loaded with Cd^2+^ showed negligible leaching after 30 days, less than 1% of total adsorbed Cd^2+^ (Table 4).

### 3.7. Removal from Various Water Matrices by Cryogels and Commercial Adsorbents

The results of Cd^2+^ removal from various water matrices and commercial adsorbents are presented in Table 5. p(MAAc)-DMAAM-AA and the commercial zeolite reached 32–35% removal of Cd^2+^ from UP water in the first 30 min, while the p(AMPS)-DMAAM removed around 28% and the ion exchange resin only 18%. After 24 h in ultrapure water the p(MAAc)-DMAAM-AA reached 88% removal in first 30 min, the commercial zeolite 77%, the p(AMPS)-DMAAM 69% and the ion exchange resin 59%. In the tap and river water, due to the presence of co-existing cations and the involvement of ion exchange mechanism as discussed in Section 2.5 [29], the removal efficiency was reduced for all materials. Nevertheless, p(MAAc)-DMAAM-AA cryogel was able to remove around 60% and 74% of Cd^2+^ from tap and river water, respectively. As in the case of ultrapure water, the commercial zeolite was more efficient than the ion exchange resin for tap and river water and reached 45% and 53% removal, respectively. The removal efficiency of the tested materials for all water matrices follows the order: p(MAAc)-DMAAM-AA > commercial zeolite > ion exchange resin > p(AMPS)-DMAAM-AA.

## 4. Conclusions

Super-macroporous cryogels were studied towards Cd^2+^ removal from aqueous solutions (ultrapure water, tap water and river water) and compared to commercial adsorbents (resin and zeolite). Owing to the functional groups and super-macroporous structure the cryogels demonstrated high adsorption capacity and fast Cd^2+^ removal kinetics. The capacity of p(MAAc)-DMAAm-AA was 239 mg/g and of p(AMPS)-DMAAm-AA 132 mg/g. The adsorption of Cd^2+^ was pH dependent and increased with the increase of the pH. At higher pH values carboxylic and sulfonate groups promote strong electrostatic interactions between the cadmium ions and the negatively charged functional groups of cryogels, which improves the adsorption capacity. XPS analysis supported the hypothesis that ion exchange followed by complexation reactions is the main mechanism of Cd^2+^ removal. In tap water p(MAAc)-DMAAm-AA removed the same amount of Cd^2+^ as the commercial zeolite and performed better than the commercial ion-exchange resin, while in river water this cryogel removed almost two times more Cd^2+^ than the commercial adsorbents. These results show that the cryogels can be attractive alternatives to commercial adsorbents, especially when fast removal is required in emergency water contamination situations. These results complement the available literature on heavy metals removal by cryogels and present novel and comprehensive data on Cd^2+^ removal by cryogels.

## Figures and Tables

**Table 1:** Polymeric materials for the removal of Cd^2+^ from the aqueous solutions.

Polymer	Initial Cd^2+^ Concentration (mg/L)	Initial pH	Metal Compound	Maximum Removal Capacity (mg/g)	Reference
GO-PEI hydrogel	100	7	CdCl_2_	175	[18]
p(AM-AMPS) hydrogel	1000	5	Cd(NO_3_)_2_	511	[6]
p(AMPS-VP) hydrogel	200	6.2	Cd(CH_3_CO_2_)_2_	70	[7]
p(HEMA-AMPS) hydrogel	1000	5	Cd(NO_3_)_2_	226	[19]
CMC-PAM hydrogel	100	5.5	Cd(NO_3_)_2_	256	[16]
NIPA-BISS hydrogel	300	6	Cd(NO_3_)_2_	130	[20]
NaAlg-g-poly(AMPS-AA-AM) hydrogel	500	7	Cd(NO_3_)_2_	457	[21]
p(AMPS-DVE-3) hydrogel	560	5	Cd(NO_3_)_2_	134	[22]
Jute/PAA hydrogel	400	6	Cd(NO_3_)_2_	402	[23]
p(GMA-co-His)	600	5	Cd(NO_3_)_2_	6.4	[24]
p(HEMA-co-MAC)	100	6	Cd(NO_3_)_2_	0.5	[25]
p(PVA-co-HA)	100	6	Cd(NO_3_)_2_	53	[26]
p(HEMA-co-VIM)/p(HEMA)	20	5.5	Cd(NO_3_)_2_	5.8	[27]
p(MAAc) cryogel	100	6	Cd(NO_3_)_2_	249	This study
p(AMPS) cryogel	100	6	Cd(NO_3_)_2_	132	

**Table 2:** Parameters of kinetic models for metal ions sorption by cryogels.

			Pseudo-First Order			Pseudo-Second Order		
	q_e_^exp^ (mg/g)	ct (mg/g)	q_e_^cal^ (mg/g)	k_1_ (1/min)	R^2^	q_e_^cal^ (mg/g)	k_2_ (g/mg×min)	R^2^
p(MAAc)-Cd	140.7	159.9	84.24	0.1622	0.977	144.9	0.0084	0.999
p(AMPS)-Cd	87.5	68.8	54.73	0.0821	0.916	98.0	0.0453	0.980

**Table 3:** Parameters of isotherm models for metal ions sorption by cryogels.

		Langmuir Model					Freundlich Model				
	q_max_^exp^ (mg/g)	q_m_ (mg/g)		K_L_ (L/mg)	R^2^	R_L_	n	K_F_		R^2^	
p(MAAc)-Cd	248.6	263.1	0.0218		0.9797	0.9978	0.105	113.4	0.817		
p(AMPS)-Cd	131.9	113.6	0.2829		0.9662	0.9725	0.121	59.4	0.873		

**Table 4:** Leaching experiment results at pH 6.5 *.

Adsorbent Type	Total Adsorbed Metal Ions (mg)	Leached Metal Ion After 30 d (mg)	Leached Metal Ion After 30 d (%)
**p(MAAc)-Cd**	10.10	0.0007	0.007
**p(AMPS)-Cd**	5.97	0.0353	0.610

* The experimental conditions: 0.07 g of material in 100 mL of 100 mg/L metal ions solution.

**Table 5:** Residual concentrations of Cd^2+^ (mg/L) in various water matrices after adsorption.

	Time (min)	30	120	1440
UP water	Ion exchange resin	16.47 ± 0.04	15.15 ± 0.30	8.35 ± 0.27
	Commercial zeolite	12.97 ± 0.72	13.39 ± 1.06	4.6 ± 0.71
	p(MAAc)	12.57 ± 0.55	12.69 ± 0.55	3.63 ± 0.03
	p(AMPS)	14.46 ± 0.85	14.01 ± 0.89	6.34 ± 0.05
	Control	20.37 ± 0.87	20.05 ± 0.11	19.88 ± 0.01
Tap water	Ion exchange resin	18.45 ± 0.21	17.71 ± 0.14	10.90 ± 0.42
	Commercial zeolite	15.45 ± 0.21	15.20 ± 0.01	7.81 ± 0.65
	p(MAAc)	15.95 ± 0.07	13.22 ± 0.01	8.05 ± 0.21
	p(AMPS)	19.50 ± 0.85	18.05 ± 0.21	15.31 ± 1.84
	Control	19.83 ± 0.23	19.50 ± 0.03	19.43 ± 0.09
River water	Ion exchange resin	15.13 ± 4.05	16.65 ± 0.78	9.40 ± 0.42
	Commercial zeolite	15.81 ± 0.28	13.25 ± 0.07	9.65 ± 0.07
	p(MAAc)	15.15 ± 0.49	14.45 ± 1.34	5.31 ± 0.14
	p(AMPS)	17.61 ± 0.57	16.05 ± 0.35	13.25 ± 2.19
	Control	19.88 ± 0.31	19.64 ± 0.67	19.54 ± 0.32

## Supplementary Materials

The following are available online at https://www.mdpi.com/2073-4360/12/10/2405/s1, Figure S1: FT-IR spectra of p(MAAc)-DMAAm-AA and p(AMPS)-DMAAm-AA cryogels; Figure S2: Graphs of surface charge determination of p(MAAc)-DMAAm-AA and p(AMPS)-DMAAm-AA cryogels at different pH; Figure S3: EDX mapping images of p(MAAc)-DMAAm-AA-Cd cryogel; Figure S4: EDX mapping images of p(AMPS)-DMAAm-AA-Cd cryogel; Figure S5: The linearized isotherm plots of p(MAAc)-Cd and p(AMPS)-Cd according to (**a,b**) Langmuir and (**c,d**) Freundlich models, respectively.
