# Supplementary material for: Removal of Cd2+ from Water by Use of Super-Macroporous Cryogels and Comparison to Commercial Adsorbents
Source: Polymers (Basel). 2020 Oct 19;12(10):2405. doi: 10.3390/polym12102405 (PMC7650616; doi:10.3390/polym12102405)
Supplement: Supplementary file 1 [file polymers-12-02405-s001.zip › polymers-957901-supplementary.docx]

*Supplementary Materials*

**Removal of Cd^2+^ from Water by Use of Super-Macroporous Cryogels and Comparison to Commercial Adsorbents**

**Alzhan Zh. Baimenov ^1,2^, Dmitriy A. Berillo ^2,3^, Seitkhan Azat ^2,4^, Talgat S. Nurgozhin ^3^ and Vassilis J. Inglezakis ^5,*^**

^1^ Environmental Science & Technology Group (ESTg), Chemical & Materials Engineering Department, School of Engineering & Digital Sciences, Nazarbayev University, Nur-Sultan, Kazakhstan; alzhan.baimenov@nu.edu.kz

^2^ Al-Farabi Kazakh National University, Almaty, Kazakhstan; berillo.d@kaznmu.kz (D.A.B.); seytkhan.azat@gmail.com (S.A.)

^3^ Kazakh National Medical University, Almaty, Kazakhstan; nurgozhin.t@kaznmu.kz

^4^ Institute of Chemical and Biological Technologies, Satbayev University, Almaty, Kazakhstan

^5^ Department of Chemical & Process Engineering, University of Strathclyde, Glasgow, UK


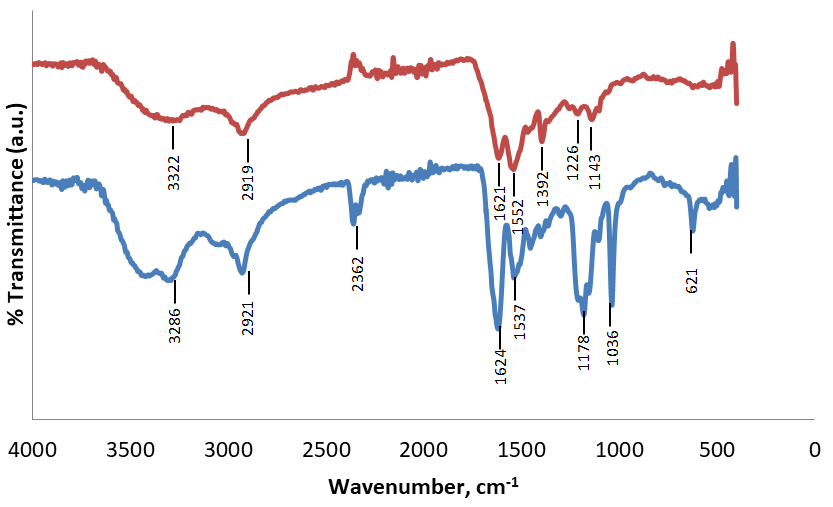


**Figure S1.** FT-IR spectra of p(MAAc)-DMAAm-AA and p(AMPS)-DMAAm-AA cryogels.

**Figure S2.** Graphs of surface charge determination of p(MAAc)-DMAAm-AA and p(AMPS)-DMAAm-AA cryogels at different pH.


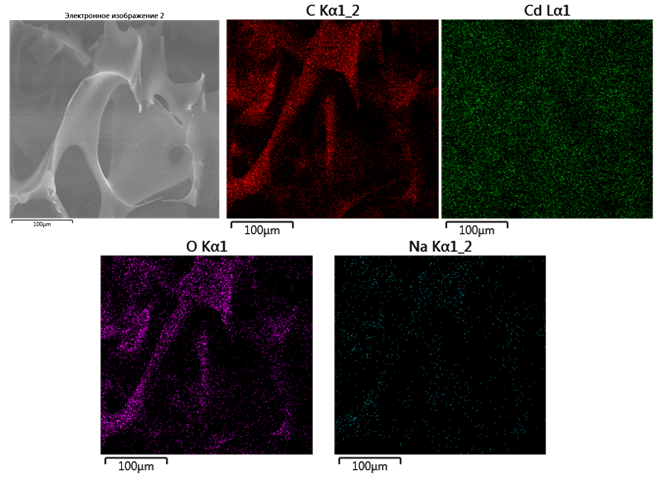


**Figure S3.** EDX mapping images of p(MAAc)-DMAAm-AA-Cd cryogel.


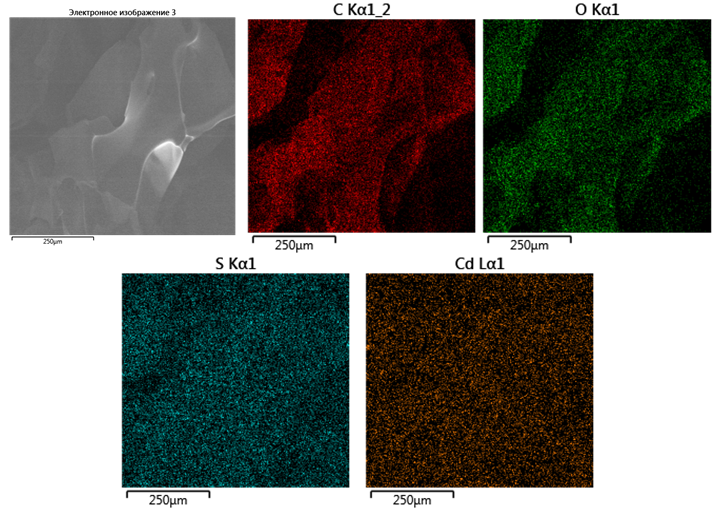


**Figure S4.** EDX mapping images of p(AMPS)-DMAAm-AA-Cd cryogel.

**Figure S5.** The linearized isotherm plots of p(MAAc)-Cd and p(AMPS)-Cd according to (**a,b**) Langmuir and (**c,d**) Freundlich models, respectively.
